# Biomass of Scyphozoan Jellyfish, and Its Spatial Association with 0-Group Fish in the Barents Sea

**DOI:** 10.1371/journal.pone.0033050

**Published:** 2012-03-22

**Authors:** Elena Eriksen, Dmitry Prozorkevich, Aleksandr Trofimov, Daniel Howell

**Affiliations:** 1 Institute of Marine Research, Bergen, Norway; 2 Polar Research Institute of Marine Fisheries and Oceanography, Murmansk, Russia; University of Wales Swansea, United Kingdom

## Abstract

An 0-group fish survey is conducted annually in the Barents Sea in order to estimate fish population abundance. Data on jellyfish by-catch have been recorded since 1980, although this dataset has never been analysed. In recent years, however, the ecological importance of jellyfish medusae has become widely recognized. In this paper the biomass of jellyfish (medusae) in 0–60 m depths is calculated for the period 1980–2010. During this period the climate changed from cold to warm, and changes in zooplankton and fish distribution and abundance were observed. This paper discusses the less well known ecosystem component; jellyfish medusae within the Phylum Cnidaria, and their spatial and temporal variation. The long term average was ca. 9×10^8^ kg, with some years showing biomasses in excess of 5×10^9^ kg. The biomasses were low during 1980s, increased during 1990s, and were highest in early 2000s with a subsequent decline. The bulk of the jellyfish were observed in the central parts of the Barents Sea, which is a core area for most 0-group fishes. Jellyfish were associated with haddock in the western area, with haddock and herring in the central and coastal area, and with capelin in the northern area of the Barents Sea. The jellyfish were present in the temperature interval 1°C<*T*<10°C, with peak densities at ca. 5.5°C, and the greatest proportion of the jellyfish occurring between 4.0–7.0°C. It seems that the ongoing warming trend may be favourable for Barents Sea jellyfish medusae; however their biomass has showed a recent moderate decline during years with record high temperatures in the Barents Sea. Jellyfish are undoubtedly an important component of the Barents Sea ecosystem, and the data presented here represent the best summary of jellyfish biomass and distribution yet published for the region.

## Introduction

It has been suggested that the abundance of gelatinous zooplankton varies considerably in many marine ecosystems around the globe and that the abundance fluctuates with climate [1], [2], [3]. Kogosek et al. [4] investigated 200 years of jellyfish data, and showed periodic jellyfish blooms in the northern Adriatic. Several anthropogenic changes capable of promoting increased jellyfish biomass have been identified, including e.g. climate change, eutrophication, pollution, overfishing, and species introductions [1], [2], . One of the main concerns is that the affected ecosystems may switch to an alternative, jellyfish dominated regime, from which it may be difficult to revert [3], [8]. Such regime shifts seem to have taken place in the Benguela current [5], [9], the Black and Caspian Seas [10], [11], and some fjords in western Norway, such as Lurefjord (e.g. [12]). However, due to the scarcity of long time series on jellyfish abundance, quantitative data verifying global increases remain scarce [2], [13], [14].

Many jellyfish are generalist predators, and often exhibit large year-to-year variations in abundance [5], [8], [15]. Major fluctuations in abundance can be linked to climatic oscillations [5], [8], [16], global warming [5] and overfishing [8], [14]. However, few long time-series from complex marine ecosystems exist. Climatic (sea surface temperature, salinity and atmospheric variability) and biological factors (e.g. density dependence, prey availability) were found to be important for the fluctuation of the abundance and distribution of scyphozoan jellyfish in the North Sea [5], [6], [8], [17], in the Irish Sea [18] and in the Bering Sea [19].

Abundant jellyfish can significantly impact the pelagic community through direct predation and competition for food (reviewed by [15], [20]–[22]), as well as through cascading effects [23]–[25]. Fish can be negatively affected through predation on fish eggs and larvae, as well as through competition for zooplankton prey (reviewed by [7], [20], [26]). Conversely, young gadoid fish (cod, haddock, Pollock, saithe and whiting) shelter among the jellyfish tentacles to avoid predation [17], [19], [27].

**Figure 1:**
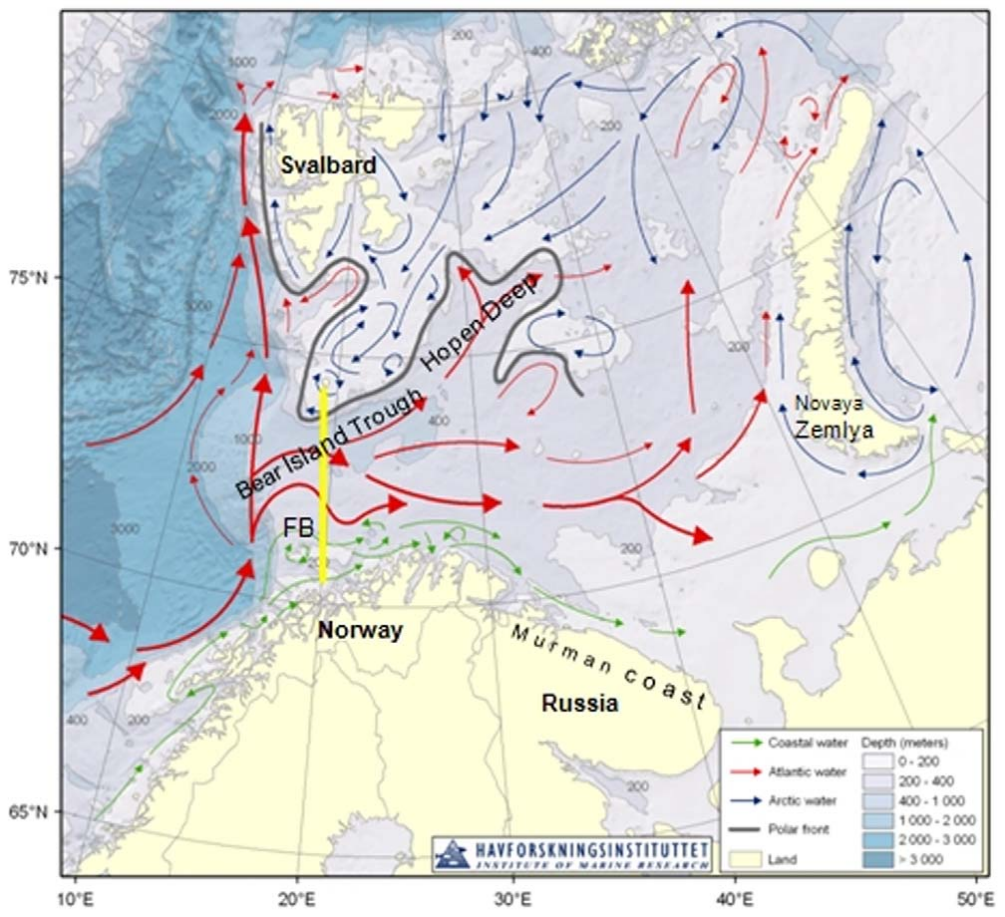
Map of the Barents Sea (www.imr.no), showing oceanographic and topographic features. The Fugløya-Bear Island (FB) section is shown by yellow line.

The Barents Sea is a high-latitude, arctoboreal shallow shelf sea, where the circulation is dominated by the Norwegian Atlantic Current entering through the Bear Island Trench in the centre of the Barents Sea (Figure 1). South of the Atlantic inflow, the extension of the Norwegian Coastal Current flows along the northern Norwegian coast and becomes the Murman Coastal Current [28]. In the northern Barents Sea, cold Arctic water generally flows south-westward [29].

The climate of the Barents Sea can be characterised as being relatively cold during the period 1900–1920 to generally warm in 1930–1950, and cold again in the late 1970s and early 1980s. The water temperature has generally increased from the late 1980s, with a peak in 2006. The inflow of Atlantic Water is of crucial importance for the physical and ecological conditions of the Barents Sea [29]. The Fugløya-Bear Island (FB) section across the western entrance to the Barents Sea is representative of the climatic variations in the Atlantic inflow [30]. The annual water temperature at 50–200 m depth from 1980 to 2008 varied between 4.6°C and 6.4°C (http://www.imr.no/sjomil). Due to the importance of the Barents Sea as a commercial fishery area and a foraging area for fish, numerous studies have been published on the fish species [31]–[36], 0-group fish [37] and mesozooplankton stocks [38], [39], [40]. Recruitment (5–8 month old fish) of commercially and ecologically important fish species, such as including Barents Sea capelin (*Mallotus villosus*), Norwegian spring spawning herring (*Clupea harengus*), Northeast Arctic cod (*Gadus morhua*) and haddock (*Melanogrammus aeglefinus*) have varied considerably between years, depending on a combination of many factors, both physical and biological [41]. In contrast, jellyfish in the Barents Sea remain poorly studied, and the overlap with 0-group fish is unknown.

**Figure 2:**
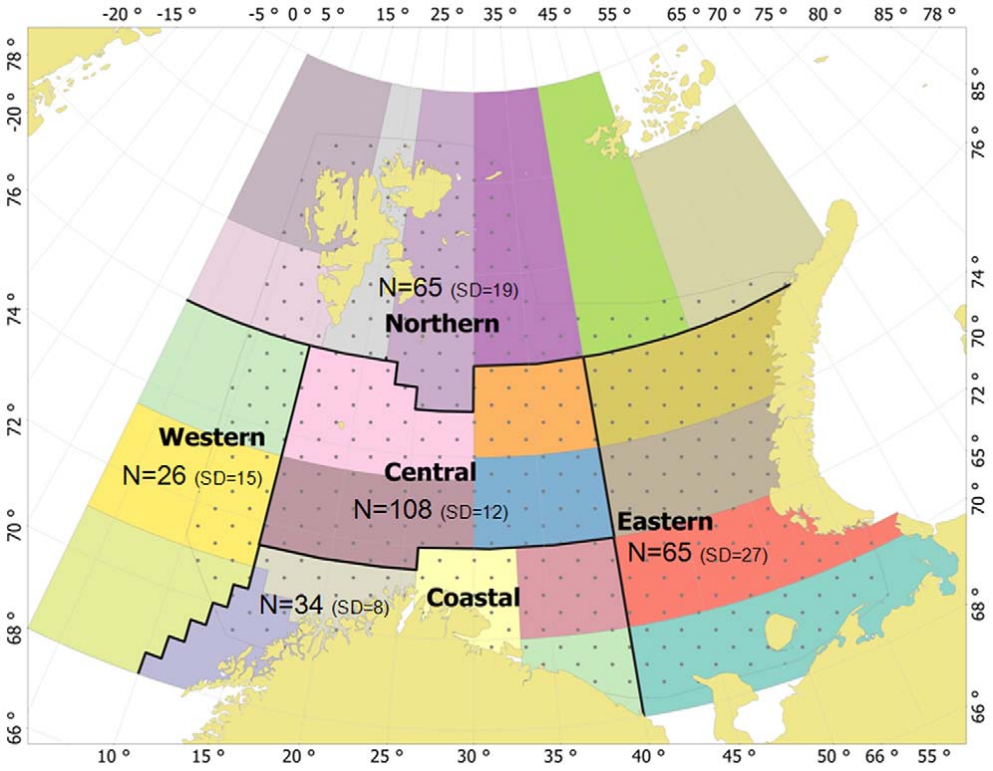
The strata system used in the jellyfish biomass calculation. The strata system is taken from that used in the 0-group fish abundance and biomass calculation [37], [50], and stratas were combined into the larger the northern, western, central, eastern and coastal areas. The 0-group survey coverage area is shown by dots. In addition, mean number of stations (N) with standard deviation (SD) per each area are shown.

In this paper, we use data from thousands of survey stations in August-September over the period 1980–2010 in the Barents Sea to explore the following questions: What is the jellyfish biomass, and how has it varied spatially and temporally? What is thermal habitat for jellyfish medusae in the Barents Sea? Furthermore, we investigate how variation in the distributions and biomasses of jellyfish medusae are related to variation in 0-group fish of capelin, haddock, cod and herring and temperature.

**Table 1:** Estimates of Barents Sea jellyfish biomass (10^6^ kg) with 95% confidence interval for the period 1980–2010.

Year	Surveyedarea, 10^9^ km^2^	Numberof stations	Mean biomass,g/m^2^	Annualbiomass, 10^6^ kg	Confidencelimit (min)	Confidence limit (max)
1980	1222	327	0.23	227	178	277
1981	1146	298	0.39	392	307	477
1982	1004	280	0.51	485	359	610
1983	1105	279	0.74	688	532	844
1984	1119	324	0.57	623	459	788
1985	1179	292	0.05	68	37	100
1986	1088	305	0.13	136	97	176
1987	1077	285	0.20	195	97	294
1988	1114	288	0.38	371	97	645
1989	1394	424	0.09	123	64	182
1990	1213	398	1.07	1279	1067	1492
1991	1312	403	0.78	973	784	1161
1992	1077	306	0.98	1096	804	1388
1993	1071	273	0.70	716	529	902
1994	952	250	0.07	63	39	87
1995	893	247	0.03	30	16	43
1996	1095	400	0.36	485	383	587
1997	948	269	0.02	19	9	28
1998	1099	361	0.21	212	169	255
1999	1040	230	0.52	524	384	664
2000	1162	269	1.07	1260	1009	1511
2001	1184	278	4.11	4906	4191	5620
2002	1129	255	2.60	2870	2436	3303
2003	1176	277	2.44	2663	2202	3125
2004	1144	309	1.33	1510	1260	1759
2005	1360	318	1.08	1423	1040	1806
2006	1078	304	1.02	1157	715	1599
2007	1297	305	1.08	1221	725	1716
2008	1246	316	0.85	1174	864	1483
2009	1274	331	0.48	664	499	828
2010	1272	304	0.23	279	359	43
**Mean**	**1144**	**307**	**0.78**	**898**		

In addition, the surveyed area (km^2^), number of stations and annual mean biomass (g/m^2^) are presented.

**Figure 3:**
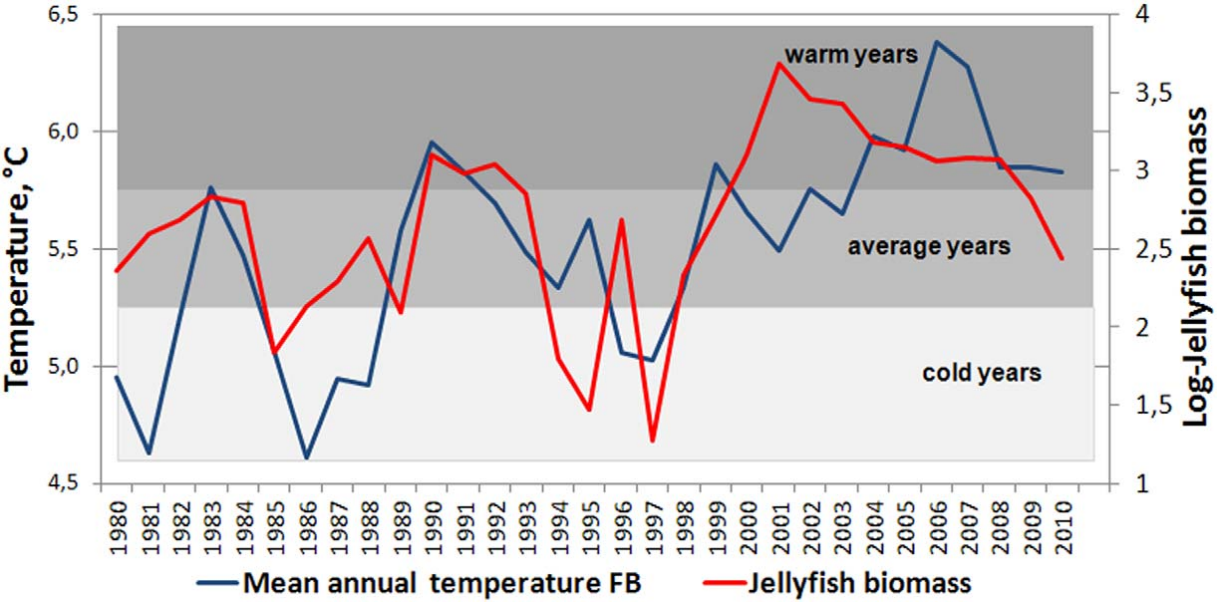
The mean annual water temperature at the Fugløya-Bear Island section (left axis) and the log transformed jellyfish biomass indices (right axis).

**Figure 4:**
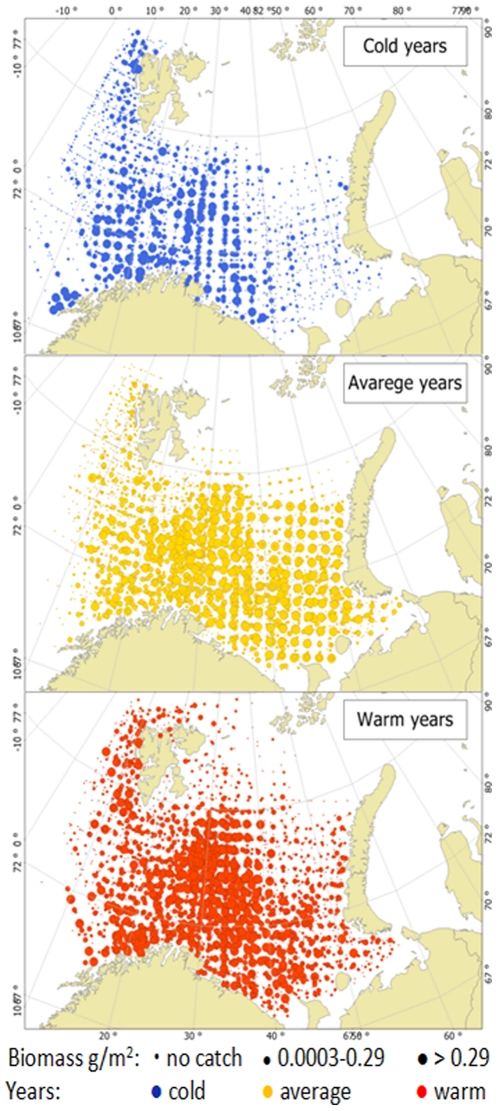
Spatial distribution of jellyfish biomass (wet weight g/m^2^) during years with different temperature regimes in the Barents Sea (see Figure 3). Cold years are shown in blue (up), average in yellow (middle) and warm years in red (bottom). Circle size indicates biomass; stations with no jellyfish are shown with the smallest circle size, 0.0003–0.29 g/m^2^ with the medium circle, and with more than 0.29 g/m^2^ jellyfish shown with the largest circle size.

**Figure 5:**
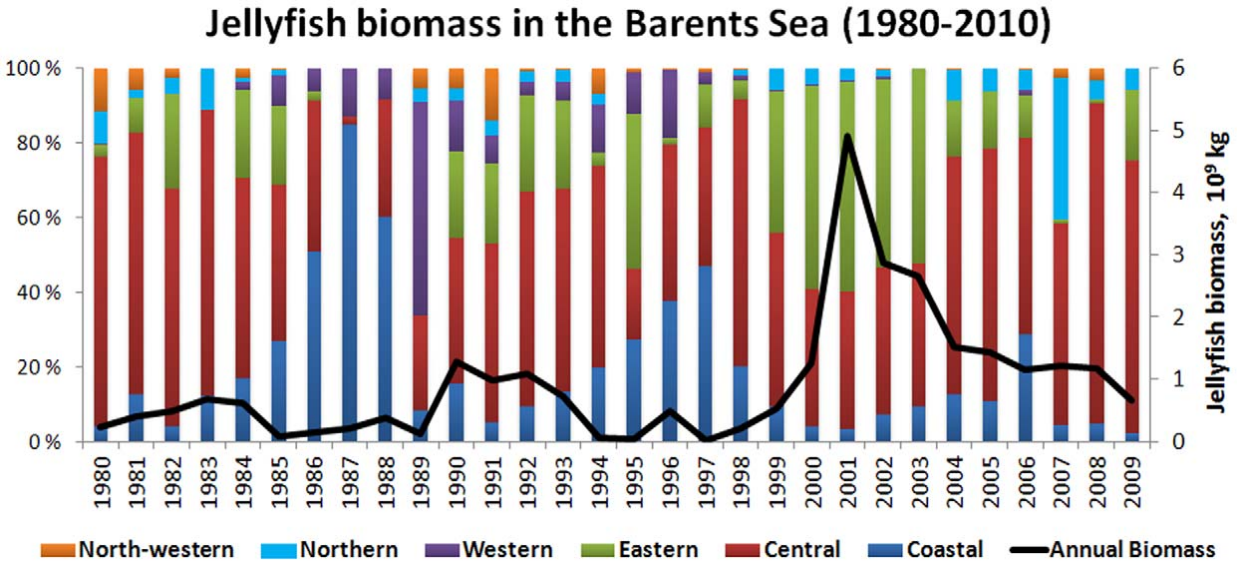
Variation of jellyfish biomass indices in the Barents Sea (10^9^ kg, black line) and the spatial distribution of jellyfish biomass (colored bars).

## Materials and Methods

### Study species

Two species of scyphozoan jellyfish commonly occur in the Barents Sea: the lion’s mane jelly *Cyanea capillata* and the moon jelly *Aurelia aurita* [42]–[44]. *Cyanea capillata* is a northern boreal species [44]–[45], while *Aurelia* is a cosmopolitan genus, particularly abundant in the coastal waters, although occurring also in the open ocean [44]. Both *C. capillata* and *A. aurita* typically exhibit large year-to-year variations in abundance [43], [46], [47]. In the Barents Sea during summer and autumn, both *C. capillata* and *A. aurita* abundance varies greatly, and their biomass or abundance indicators give early warning signals with respect to climate change [43]. In the Northeast Atlantic strobilation (asexual budding of jellyfish ephyrae from benthic polyps) of *C. capillata* takes place in the late winter and spring [46], [48], although there seems to be differences in the timing of reproductive events between populations [7], [47], [49]. The timing of reproductive events in the Barents Sea area is currently not known.

### Survey

A Joint Norwegian-Russian 0-group survey has been carried out annually in August-September in 1965–2003. Since 2004 the 0-group investigations have continued as part of a Joint Norwegian-Russian ecosystem survey (here referred to as O-group survey). The survey provides data for the estimation of 0-group fish abundance indices for the most important commercial fish species. By-catch, including jellyfish medusae within the Phylum Cnidaria, (hereafter call jellyfish), has only been weighed and not identified to species level. The trawling procedure consists of tows on predetermined positions 46–65 km apart along the survey track. At each station a pelagic “Harstad” trawl is towed at 3 or more depths, with the head-line at 0 m, 20 m and 40 m depths and each depth tow of ca 900 m with a trawling speed of 5.56 km/h. Additional tows at 60 and 80 m, also of ca 900 m, were made when dense fish concentrations were recorded deeper than 40 m depth on the echo-sounder, but the number of such deeper stations is low. The pelagic “Harstad trawl” has a 20 m×20 m mouth opening, and consists of 7 panels and a cod end. The mesh size varied from 100 mm in the first panel to 30 mm in the last. The cod end consisted of a 30 m long capelin net with 20 mm meshes for catching pelagic fish, and a 14 m long inner net with 7 mm meshes for catching 0-group fish. Therefore, we believe that larger *C. capillata* may be captured by all panels, while smaller and less robust species, such as *A. aurita* are also probably sieved through trawl meshes. It is likely they are only reliably captured by the last panel, and probably partially or totally destroyed in the cod end.

The joint Norwegian-Russian fish database has recently been corrected and updated for the period 1980–2006 [50]. The data for jellyfish were missing from the electronic database; therefore, the first task of the present study was to update the database for the period 1980–2010 to include jellyfish data. These data have not been previously analysed and published.

### Biotic data

#### Jellyfish

Data for scyphozoan jellyfish were collected from pelagic trawl catches during the 0-group survey in the Barents Sea. Over the study period (1980–2010) 9529 pelagic trawl stations, each with 3 trawl depths or more, were sampled. We used these data to estimate biomass indices of jellyfish in the Barents Sea for the period 1980–2010, and to examine spatial overlap with 0-group fish for the shorter period (1980–2008) due to missing temperature data in the database.

#### 0-group fish (cod, haddock, herring and capelin)

Fish data were collected from pelagic catches during the 0-group survey in the Barents Sea (1980–2008). We used these data to calculate fish density (individuals per m^2^) for each trawl haul with regard to catch and trawl haul data (depth interval, effective opening and distance trawled). The method is described by Dingsør [51] and Eriksen et al. [50]. Eriksen et al. [41] described several areas in the Barents Sea (the coastal, central, eastern, western, north-western and northern), based mostly on bathymetric and water features. To make our results comparable with this early study we use a similar spatial division (Figure 1), except for the north-western and northern areas, which we combined due to limited fish and jellyfish catches there. This combined area we hereafter call the northern area.

### Abiotic data

The water temperature data are from CTD (Conductivity, Temperature and Depth sensors) samples taken at each 0-group trawl station. The CTD profiles were taken either before or after trawling, and in this study we used the temperatures aggregated to standard depths (5 m, 10 m, 20 m, 30 m, 40 m). Over the study period (1980–2008), 7089 CTD stations were conducted. We used these data to define temperature ranges for jellyfish.

Temperature (and since 1997, volume inflow) of Atlantic Water to the Barents Sea has been measured monthly at the standard oceanographic section Fugløya-Bear Island (70°30′ and 20°00′ to 74°15′ and 19°10′, Figure 1) by the Institute of Marine Research (IMR, Norway,). The water temperature was measured by CTD at standard depths at predetermined stations along the FB. Here we use a time series of annual temperature at 50–200 m depth taken from the path of Atlantic inflow. The annual mean temperature from 1980 to 2008 was 5.5°C, and years were categorized into three similar groups: average (long term mean temperature ±16% of the long term mean value), cold (below average) and warm (above average).

### Data treatment

We calculated the following:

#### Biomass indices

Biomass indices for the period 1980–2010 using the stratified sample mean method of swept area estimates [51]. For jellyfish biomass estimation, the Barents Sea 0-group strata system, which consists of 23 strata, was used (Figure 2). The biomass (g/m^2^), *b_s_*, at each station, *s*, was estimated by the equation
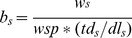
(1)


where *w_s_* is the catch (g) at station *s*, *wsp* is the effective wingspread of the trawl (20 m), 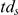
(m) the total distance trawled at station *s*, and 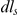 is the number of depth layers at station *s*. If the number of depth layers at station, *s*, is 1, it means that the trawl was towed for ca 900 m at the surface (0 meter depth) covering the water layer between 0 and 20 m. If the number of depth layers at station, *s*, is 2, it means that trawl was towed for ca 900 m covering 0–20 m and ca 900 m at 20–40 m, and so on.

For each of the strata the total biomass, *B,* was calculated by
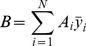
(2)


where *N* is the number of strata, *A_i_* is the area covered in the *i*-th stratum, and 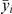 is the average biomass in stratum *i* given by
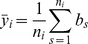
(3)


where *n_i_* is the number of stations in stratum *i,* and *b_s_* is biomass (g/m^2^), at each station, *s.*

The estimated variance of the B is given by
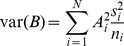
(4)


where
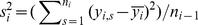
(5)


A biomass estimate (10^9^ kg) for each area (the coastal, central, eastern, western, and northern) was calculated as the sum of the stratified swept area biomass estimates (*B*) of all strata within the area (see Figure 2). Jellyfish biomasses were not interpolated to the whole strata/area, and represent only the covered area. The estimated jellyfish biomass is likely to be conservative, since smaller individuals may have passed through the larger mesh sizes, and some of jellyfish species distribute deeper than the sampled depths (0–60 m) or outside the covered area. Consequently, the estimated biomasses may be interpreted as minimum biomass.

#### The fish density

The fish density **(**individuals/m^2^) for each trawl haul was calculated using catch and trawl data (depth intervals, effective opening and distance trawled). The method is further described by Dingsør [51] and Eriksen et al. [50].

#### The jellyfish biomass

The jellyfish biomass**,** wet mass (g/m^2^), for each trawl haul was calculated with regard to catch and trawl data (depths interval, effective opening and distance trawled). The method is further described by Dingsør [51] and Eriksen et al. [50].

#### The mean temperature

The mean temperature per station for the water layer 5–50 m calculated as the average of the temperatures from standard depths. Temperature was recorded at a total of 7089 CTD stations.

### Statistical modelling

We investigated relationships between jellyfish, 0-group fish of cod, haddock, herring and capelin, and temperature for the period 1980–2008. We used a GAM (Generalized Additive Model) in the R (version 2.12.2) package mgcv [52]. To study associations between the biomass of jellyfish (JF_B_) and densities of 0-group capelin (Cap_D_), haddock (Had_D_), cod (Cod_D_), herring (Her_D_) and temperature in sample (i.e. station) *i* were fitted to the covariate according to the following models:
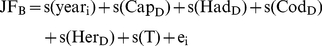
(6)


where the additive effect included the smoothed fits (s) of variables of sample i. Year was included as a factor in the model (Equation 6), e_i_ denotes the error for sample *i*. Separate models were constructed for the coastal, central, eastern, western, and northern areas. We used backward selection to identify the best model based on Akaike information criterion (AIC) and genuine cross validation (GCV).

#### Core Thermal Habitat (CTH)

A temperature range was estimated from the model as the temperatures corresponding to jellyfish biomasses larger than the mean modelled jellyfish biomass. This temperature range, including about ∼60% of the observations we hereafter call the Core Thermal Habitat (CTH).

## Results

The estimated jellyfish biomass the Barents Sea varied considerably from year to year (Table 1 and Figure 3). Jellyfish biomass was generally low during the 1980s, moderately high in the 1990s, and high in 2000s, and the mean biomass (10^6^ kg) was about 330 *(SE = 68; SD = 216)*, 540 *(SE = 147; SD  = 465)* and 1700 *(SE = 390; SD  = 1295)* respectively. Estimated jellyfish biomass varied from 19×10^6^ in 1997 to ca. 5×10^9^ kg in 2001, with a long term mean for the period 1980–2010 of around 1×10^9^ kg *(SE = 184; SD = 1023)*. The long term mean biomass of jellyfish was approximately 0.78 g/m^2^ *(SE = 0.16; SD = 0.87)*. The highest biomasses occurred during 2001–2003, when mean biomass was 2.4–4.1 g/m^2^ and station specific biomass ranged as high as 44.3 g/m^2^. There has been a decrease in biomass since 2009 (Figure 3).

The spatial distribution of jellyfish biomass varied between years with different temperature conditions. The most restricted distribution and generally low catches were observed during cold years, while during average and warm years jellyfish occupied almost whole of the Barents Sea, and catches were very high (Figure 4). Jellyfish biomass also varied between different areas in the Barents Sea. The central and eastern areas contributed most of the total jellyfish biomass. Their average contribution over the study period was about 49% and 31% respectively (Figure 5), although the proportion of jellyfish in these areas showed considerable variability. The eastern area, in particular, showed highly varied levels of jellyfish biomass. A high proportion of the total biomass was found in the eastern area during years with unusually high total biomasses (1999–2003 and to a lesser extent 1990–1993), whereas in the lowest years (1986–1989) there was little or no jellyfish reported from the eastern area (Figure 5). In years with low estimated biomass the relative importance of the coastal area increased, although the average jellyfish density in the coastal area over the study period was approximately half of that in the central area, at 81.6 g/m^2^ in the coastal region compared with 131.2 g/m^2^ in the central area. Averaged over the whole period (1980–2010), the contribution from the coastal, western and northern areas were low, and these areas contribute only 11%, 2% and 7% of total jellyfish biomass, respectively. However, the coastal area contained a relatively stable population, in contrast to the marked variability in the other areas (Figure 5).

**Figure 6:**
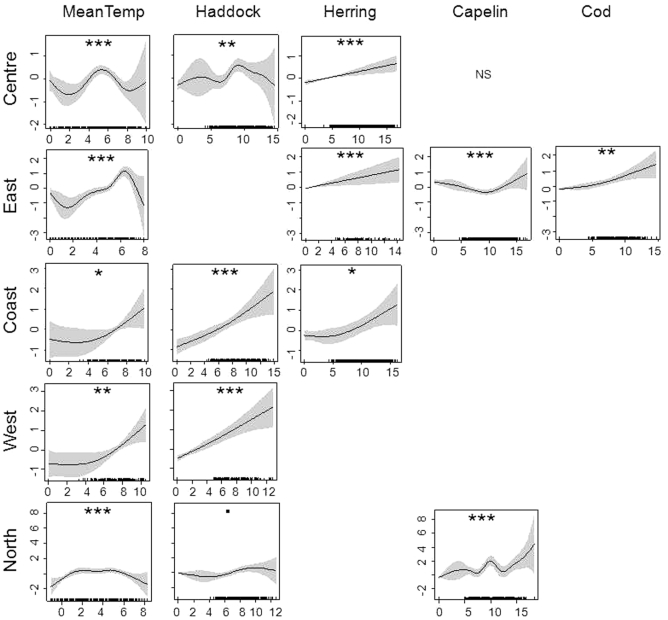
Estimated functions for jellyfish and prognostic factors (mean temperature (MeanTemp) and 0-group fish (haddock, herring, capelin and cod) densities). Jellyfish biomass shows at y-axis, while prognostic factors at x-axis. Separate models were performed for the coastal, central, eastern, western, and northern areas codes are shown: 0.001 as “***”, 0.01 as”**”, 0.05 as ”*”, 0.1 as”^.^”, and not significant means as Ns.

Jellyfish were associated with 0-group cod, haddock, herring and capelin in the areas where fish were abundant, and these relationships varied between areas (Table 2, Figure 6). Jellyfish biomass was positively correlated with haddock (coastal and western areas) and herring (central, eastern and coastal area) and cod (eastern area). In the central area we found no association between jellyfish and 0-group cod, and the association with haddock was non-linear. In the northern area, dominated by 0-goup capelin, jellyfish was associated only with capelin, and the association was non-linear.

**Table 2:** Additive models for the relationship between jellyfish, temperature, haddock, cod herring and capelin in the different areas in the in the Barents Sea, adjusted R^2^ (i.e. proportion of variance explained), and genuine cross validation (GCV).

Models	s/F Mean Temp	s/F Haddock	s/F Herring	s/F Capelin	s/F Cod	R^2^	GCV score/Scale est.
Central	4.95/4.45	5.06/3.46	1.03/11.84	ns		0.37 (37.7)	10.18 (10.02)
East	6.36/8.34		1.00/7.32	3.00/5.62	1.76/8.86	0.55 (56.2)	6.84 (6.68)
Coastal	1.98/4.05	1.3/11.00	1.87/3.51			0.24 (27.2)	11.78 (11.25)
Western	1.93/5.50	1.18/21.28				0.32 (35.1)	7.59 (7.17)
Northern	4.09/5.02	3.03/2.13		6.9/5.02		0.25 (27.7)	9.13 (8.82)

The selected model includes both significant terms (i.e. p≤ 005) and not significant terms (i.e. p≤ 0.05).

The jellyfish were present in the temperature interval 1°C<T<10°C (Figure 6). The CTH, was bounded in a temperature band around the maximum between 4.0–7.0°C, indicating that jellyfish associated with Atlantic water masses. However, the jellyfish showed a dome shaped distribution with temperature in the central and coastal areas, with maximum biomass at ca. 5.5°C and 6.5°C, respectively (Figure 6). In the coastal and western area, jellyfish were positively correlated with temperature (Figure 6).

## Discussion

The Barents Sea is a productive ecosystem, with more than 200 fish species, thousands of benthic invertebrate species, and diverse communities of plankton, seabirds and marine mammals which inhabit or visit the area [53]. Only a few fish species, including cod, haddock, saithe, capelin, redfish, Greenland halibut, and polar cod are of commercial interest. Nonetheless, these form the basis of one of the largest fisheries in the world [54]. Historically, scientific surveys focused on monitoring commercially important fish stocks, although after a strong decrease of the cod fishery in the Barents Sea, and a near collapse in herring fishery in the Norwegian Sea, an 0-group fish survey was started in order to give early signals of fish recruitment and further stock development. Gelatinous zooplankton have not been the focus of research until recent decades. Recent trends, including some of the old fish recruitment “rules of thumb” ceasing to apply, a strong increase of the temperature in the Barents Sea [41], [50], and an increasing focus on the impacts of rising populations gelatinous zooplankton in other ecosystems [1], [2], [5]–[7] has changed this. Understanding jellyfish distributions and interactions with other species is increasingly seen as important in order to avoid having a “black box” in our understanding of the ecosystem. In order to gain an insight into jellyfish presence and especially their relationship with 0-group fish, we have used available trawl data from 0-group fish survey.

Sampling jellyfish is problematic, due to an extremely patchy distribution and fragile nature, making both standard fisheries gear and conventional plankton nets of limited value [55]. Several studies have used by-catch of jellyfish from fisheries surveys [14], [56], [57], but the large mesh size of the gear typically used in such surveys is not well suited to catching jellyfish. Our data were collected by small “Harstad” trawl with small mesh size (see above). This trawl is smaller than standard fisheries trawls gear used in previous studies, and therefore has increased catchability and decreased chance of damage to jellyfish within the trawl net. This sampling gear is also larger than conventional plankton nets and therefore i) better able catch larger jellyfish, *C. capillata,* due to larger effective opening and faster trawling speed, but ii) less able to catch smaller jellyfish, *A. aurita*, which is sieved through trawl meshes or partially or totally destroyed in the cod end [53]. Therefore, our results consist mostly of *C.capillata*, and the biomass presented must be interpreted as minimum for the total jellyfish biomass in the Barents Sea. Nonetheless, long term monitoring of the Barents Sea using this standard sampling procedure and standard regular station grid makes data comparable between vessels, areas and years.

The biomass of Barents Sea jellyfish varied considerable between years and higher jellyfish biomasses were generally found in the beginning of 1990s, and high or record high during the 2000s, with a moderate decrease in the end of 2000s (Table 1, Figure 5). In the Bering Sea an increase of jellyfish catches was observed during the 1990s, with a maximum in 2000, moderate amounts during period 2001–2007, and decreased amount in 2008 [57]. Both Lynam et al. [18] and Brodeur [57] found that a warming trend favouring many species of jellyfish in other seas. During the period studied here, temperature conditions changed from cold during the 1980s, to moderate in the 1990s, and to warm during the last decade. It seems that, at least up to a certain point, a warming trend is also favourable for the Barents Sea jellyfish. Warmer temperature conditions in the Barents Sea are associated with increased inflow of Atlantic water, bringing more zooplankton from the Norwegian Sea into the Barents Sea [58] and better feeding conditions for plankton feeders from larvae to adult [58], [59], [60]. The highest biomasses of jellyfish were found in the temperature range of 4–7.0°C, indicating that i) an increase of temperature may not lead to further increases in jellyfish biomass in the Barents Sea, and ii) the greater proportion of jellyfish are resident in water masses of Atlantic origin (i.e. waters having temperatures above 3°C, [61]), with a lesser proportion distributed in the mixed water masses (i.e. waters having temperatures between 0°C and 3°C [61]). Spatial distribution of jellyfish varied between years and was widest during the 2000s (Figure 4). The greater proportion of jellyfish occurred in the central area throughout the time series. The highest plankton biomass was observed during the summer at the entry of the Barents Sea due to the ocean currents, making this area the core nursery area for 0-group fish [41], [62]. In the central area, jellyfish overlapped mostly with cod, haddock and herring, although a statistically significant relationship was only found with haddock. This relationship was dome-shaped, with low jellyfish biomasses where there was a low or high density of fish, while the highest jellyfish biomasses overlapped with averaged values of haddock. During the 2000s, a substantial increase in areas with mixed water has been observed in the Barents Sea [61], and such redistribution of water masses seems likely to impact the jellyfish distribution by extending of the area with suitable living conditions (Figure 4). A similar redistribution has been observed for 0-group capelin, suggesting sufficient feeding conditions in the eastern and northern areas [41]. Our results showed significant overlap between jellyfish and 0-group capelin in the eastern and northern areas, however jellyfish biomass and 0-group density were much lower than in the central area and varied considerable between years.

In contrast, the coastal areas acted as a reserve area for the jellyfish, with relatively constant biomasses over time. Scyphozoan jellyfish success in the coastal waters is determinate by morphological, behavioural and life history characteristics [63]. This rich coastal environment seems to have led to a stable jellyfish population in this area, resulting in it forming a significant fraction of the population during low biomass years. In the coastal area jellyfish significantly overlapped with 0-group of haddock and herring, and higher biomasses of jellyfish were observed with higher fish densities and increasing temperature. The complex physical structure of coast supports zooplankton productivity [64], [65], [66], and may impact positively on amount of jellyfish and 0-group fish there.

During years with extremely high amounts of jellyfish (2001–2003), no strong fish year classes occurred. These years were characterized by average or high spawning stock biomass of cod, haddock, herring and capelin (except cod, in 2001 SSB was lower than average) and warmer temperature conditions as proxy for better feeding conditions and successful recruitment of cod, haddock and herring. One might therefore expect the occurrence of average or strong year classes during this period. It seems that jellyfish was positively related with 0-group herring (the central and coastal areas), cod (eastern), and 0-group haddock (western), indicating that they inhabited similar water masses. It is possible, therefore, that the large stock of biomass played a role in preventing the occurrence of large year classes during this period. However, the relationship between jellyfish and 0-group fishes is complex and depends on many factors both physical and biological (reviewed by [15], [20], [21], [22]), making it difficult to separate influence of different factors and combination of them. Thus, diet studies of both 0-group fish and jellyfish are needed to understand spatial overlap between them, and we recommend to prioritize species identification of jellyfishes onboard during this survey to minimize uncertainties surround the biomass indices calculation.

The Barents Sea is an important commercial fishery area, currently containing the largest cod and capelin stocks in the world, and in 2010 the fish and shrimp catches were reported to be close to 2.9×10^9^ kg [67]–[69]. Marine mammals are also harvested, although on a smaller scale. Removal of top-predators such as demersal fish through fisheries might cause trophic cascades and abrupt changes in ecosystem state [70]. Despite a high level of exploitation of demersal and pelagic fish, high jellyfish biomass (such as 5×10^9^ kg in 2001) and a trend of increasing temperatures, no dramatic shifts have been reported from the Barents Sea. However, many of the long established relationships and mechanisms in the Barents Sea seem to be changing.

This study provides i) basic information about the spatial and temporal distributions of jellyfish biomass in the Barents Sea, ii) indicates the complexity of an ecosystem including jellyfish, rather simple ecological effect on 0-group fish or whole system, and iii) suggests a possible jellyfish core temperature habitat in the Barents Sea. This study is based on long term (and ongoing) monitoring, and gives a insight into the Barents Sea ecosystem which may be useful for ecosystem modellers, researches within plankton, ecology and fisheries biology and fisheries managers around the world.
